# Multifunctional polymeric nanoparticles doubly loaded with SPION and ceftiofur retain their physical and biological properties

**DOI:** 10.1186/s12951-015-0077-5

**Published:** 2015-02-13

**Authors:** Paula Solar, Guillermo González, Cristian Vilos, Natalia Herrera, Natalia Juica, Mabel Moreno, Felipe Simon, Luis Velásquez

**Affiliations:** Universidad Andres Bello, Facultad de Medicina, Center for Integrative Medicine and Innovative Science, Echaurren 183, Santiago, Chile; Departamento de Ciencias y Tecnología Farmacéutica, Universidad de Chile, Facultad de Ciencias Químicas y Farmacéuticas, Santos Dumont 964, Independencia, Santiago, Chile; Center for the Development of Nanoscience and Nanotechnology, CEDENNA, 9170124, Av. Ecuador 3493, Estación Central, Santiago, Chile; Departamento de Química, Laboratorio de Síntesis Inorgánica y Electroquímica, Universidad de Chile, Facultad de Ciencias, Las Palmeras 3425, Nuñoa, Santiago, Chile; Departamento de Ciencias Biológicas, Facultad de Ciencias Biológicas, Universidad Andres Bello, República 252, Santiago, Chile; Facultad de Medicina, Universidad Andres Bello, República 590, Santiago, Chile; Millennium Institute on Immunology and Immunotherapy, Avenida Libertador Bernardo O’Higgins 340, Santiago, Chile

**Keywords:** PHBV, SPION, Ceftiofur, Polymeric nanoparticles, Drug delivery, Superparamagnetic nanoparticles

## Abstract

**Background:**

Advances in nanostructure materials are leading to novel strategies for drug delivery and targeting, contrast media for magnetic resonance imaging (MRI), agents for hyperthermia and nanocarriers. Superparamagnetic iron oxide nanoparticles (SPIONs) are useful for all of these applications, and in drug-release systems, SPIONs allow for the localization, direction and concentration of drugs, providing a broad range of therapeutic applications. In this work, we developed and characterized polymeric nanoparticles based on poly (3-hydroxybutyric acid-co-hydroxyvaleric acid) (PHBV) functionalized with SPIONs and/or the antibiotic ceftiofur. These nanoparticles can be used in multiple biomedical applications, and the hybrid SPION–ceftiofur nanoparticles (PHBV/SPION/CEF) can serve as a multifunctional platform for the diagnosis and treatment of cancer and its associated bacterial infections.

**Results:**

Morphological examination using transmission electron microscopy (TEM) showed nanoparticles with a spherical shape and a core-shell structure. The particle size was evaluated using dynamic light scattering (DLS), which revealed a diameter of 243.0 ± 17 nm. The efficiency of encapsulation (45.5 ± 0.6% w/v) of these polymeric nanoparticles was high, and their components were evaluated using spectroscopy. UV–VIS, FTIR and DSC showed that all of the nanoparticles contained the desired components, and these compounds interacted to form a nanocomposite. Using the agar diffusion method and live/dead bacterial viability assays, we demonstrated that these nanoparticles have antimicrobial properties against *Escherichia coli*, and they retain their magnetic properties as measured using a vibrating sample magnetometer (VSM). Cytotoxicity was assessed in HepG2 cells using live/dead viability assays and MTS, and these assays showed low cytotoxicity with IC_50_ > 10 mg/mL nanoparticles.

**Conclusions:**

Our results indicate that hybrid and multifunctional PHBV/SPION/CEF nanoparticles are suitable as a superparamagnetic drug delivery system that can guide, concentrate and site–specifically release drugs with antibacterial activity.

**Electronic supplementary material:**

The online version of this article (doi:10.1186/s12951-015-0077-5) contains supplementary material, which is available to authorized users.

## Background

Superparamagnetic nanoparticles are used in multiple biomedical applications, such as for contrast medium in magnetic resonance imaging (MRI)-based diagnosis, in hyperthermia applications and for tissue-specific drug delivery using an external magnetic field in cancer treatment [1]. Hyperthermia is a treatment were the target tissue is exposed to temperatures slightly higher than physiological to damage and kill cells.

There are many different superparamagnetic nanoparticles; however, superparamagnetic iron oxide nanoparticles (SPIONs) are currently the best option for biomedicine because they are biocompatible with the body. Actually, for diagnostics, gadolinium salts (Gd-DTPA) are the gold standard contrast medium for MRI, but their resolution and retention time in vivo remains low (2 mm for MRI) [2-4]. However, Gd-DTPA have been linked to anaphylactic reactions and toxicity at the hepatic and renal levels. Therefore, it is important to find alternative contrast media that have low toxicity and better resolution for MRI. In this field, SPIONs function as a more sensitive contrast medium; the obtained images have better resolution, and the particles have a better retention time *in vivo* and are more biocompatible than Gd-DTPA [4]. The use of a SPION-like contrast medium for MRI is not the only potential application of SPIONs. Theoretically, SPIONs can be used for multiple actions, thus generating multifunctional nanoparticles. SPIONs can be used for hyperthermia applications and drug delivery [5], allowing for the localized and controlled release of drugs.

The application of nanoparticles *in vivo* depends on their physicochemical properties such as their hydrophobicity, net surface charge and size. These properties have a direct impact on their biocompatibility, biostability and toxicity. The use of SPIONs is limited because SPIONs are not stable in physiological environments due to their very reactive surface. SPIONs liberate Fe^2+^ ions, produce oxidative stress via the release of reactive oxygen species (ROS), and alter ion transport [6]. Lastly, SPIONs are not stable in aqueous media because they tend to agglomerate and precipitate [4]. To overcome these limitations, we have proposed coating SPIONs with biocompatible materials to preserve all of their potential uses while preventing their toxicity [7].

The development of nanotechnologies that utilize biocompatible and biodegradable polymers has provided new tools for diagnostic and therapeutic strategies in biomedicine [8], wherein one of the most important areas is drug delivery. Current novel therapeutic strategies include the delivery of cell tissue-specific drugs, the use of agents that allow for the imaging of release sites [9], delivery systems that can cross epithelial and endothelial barriers [10], the intracellular delivery of macromolecules, improvements in drugs with poor water solubility, the co-release of therapeutic agents, and an improvement in effective therapies [11,12]. For these applications, a biocompatible and biodegradable polymer is the best candidate for a SPION coating because this type of coating may decrease their natural reactivity and maintain their physical properties. Poly (3-hydroxybutyrate-co-3-hydroxyvalerate) (PHBV), a polymer that has recently been used for drug delivery and tissue engineering [13], is cost effective and has physicochemical properties similar to those of the most widely used polymers such as poly(L-lactide) [14], poly(D,L-lactide-co-glycolide) [15-18], poly-sebacic anhydride [19] and poly-ε-caprolactone [20-22].

One aim in drug delivery is to transport antibiotics. In this area, we determined that PHBV can be used to encapsulate and deliver the antibiotic ceftiofur in microparticles [23,24] and PHBV can interact with β-lactams to form a new microcomposite with specific physicochemical properties. We analyzed the same antibiotic that was previously assayed (ceftiofur), and we incorporated SPIONs into PHBV nanoparticles to generate a new nanocomposite. Furthermore, we analyzed the molecular structure and the cytotoxicity of PHBV nanoparticles loaded with SPIONs and ceftiofur (PHBV/CEF/SPION); for controls, we used each component and nanoparticles with different combinations of their components: PHBV nanoparticles with only ceftiofur (PHBV/CEF) and PHBV nanoparticles with only SPIONs (PHBV/SPION).

Due to the potential uses of SPIONs and the advantages of polymeric nanoparticles, PHBV/CEF/SPION nanoparticles could (i) be used as a nanocarrier for tissue-specific drug delivery, (ii) allow for MRI-based diagnostics and for therapy using their antibacterial activity or hyperthermia using only one injection, and (iii) be used as a multifunctional treatment for hyperthermia and for the release of antibiotics at an infection site.

## Results and discussion

**Figure 1:**
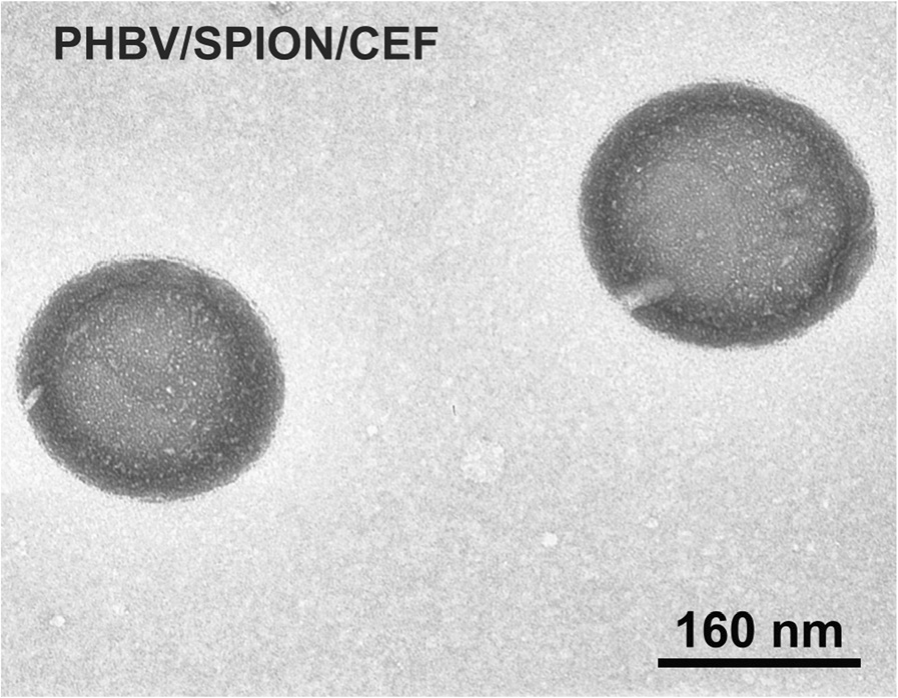
**Representative image of PHBV/SPION/CEF obtained by transmission electron microscopy (TEM).** Bar: 160 nm.

TEM images (Figure 1) show PHBV/CEF/SPION nanoparticles with a core-shell structure, a spherical shape, a smooth surface and a moderately uniform size distribution. This image shows nanoparticles with dense black spots inside. The mean size (diameter, nm) of the formulated PHBV/CEF/SPION measured using DLS was 243.0 ± 17.

**Figure 2:**
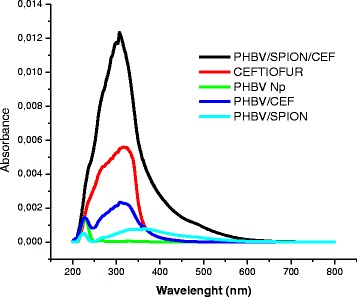
**UV–VIS spectroscopy of PHBV nanoparticles and ceftiofur.**

To determine the amount of ceftiofur that was able to incorporate into the PHBV nanoparticles, we analyzed the individual components of these lyophilized nanoparticles using UV-visible (UV–VIS) spectroscopy (Figure 2). The UV–VIS spectra of nanoparticles with different combinations of components are shown: PHBV nanoparticles with ceftiofur, PHBV nanoparticles with SPION and PHBV nanoparticles with SPION and ceftiofur. For controls, we used empty PHBV nanoparticles and free ceftiofur. Analysis of the ceftiofur spectrum (Figure 2, red line) shows an intense broad band centered at approximately 320 nm. The deconvolution of the signal at 320 nm showed that it mainly corresponds to the superposition of four peaks, namely, two low-intensity absorption peaks centered at 235 and 294 nm and two more intense absorption peaks centered at 265 and 325 nm. The lower energy and higher intensity of the latter absorbances correspond to π-π* transitions for a highly delocalized system. Because of the molecular structure of ceftiofur, this absorption band could be assigned to the polyenone arising from the alkyloximino C = N- chromophore modified by the thiazolyl moiety. The peak observed at 294 nm with lower intensity could correspond to the C = O chromophore from the amide group, which is also near the thiazolyl group. The high intensity band at 265 nm could also be assigned to π-π* transitions centered in the furan-carboxylic-thioester, whereas the lower intensity peak at higher energy (or lower wavelength) most likely arises from the C = O lactam moiety. Absorption by the carboxylic acid-dihydrothiazine chromophore is expected to occur at higher energies, and thus it was not observed in the spectrum.

PHBV nanoparticles could potentially act as a nanocarrier that incorporates ceftiofur; thus, we evaluated the encapsulation efficiency (EE%) of ceftiofur. The EE% of lyophilized nanoparticles, measured using Ultra performance liquid chromatography (UPLC) and UV–VIS absorbance at 302 nm, was compared with a previously set calibration curve. The PHBV/CEF nanoparticles contain 11.41 wt% ceftiofur, and the PHBV/CEF/SPION nanoparticles contain 11.43 wt% ceftiofur. As the SPIONs produce high levels of noise in the UV–VIS spectrum, we corroborated this result using UPLC with three independent samples. The %EE of ceftiofur in PHBV nanoparticles was 45.5 ± 0.6.

**Figure 3:**
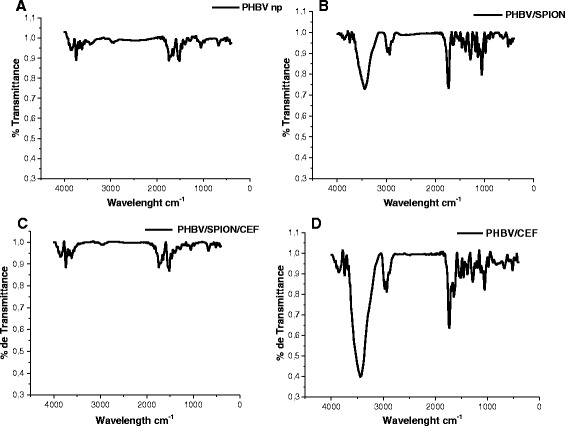
**FTIR spectroscopy of PHBV nanoparticles. A)** empty PHBV nanoparticles, **B)** PHBV/SPION nanoparticles, **C)** PHBV/SPION/CEF nanoparticles and **D)** PHBV/CEF nanoparticles. The controls, FTIR of ceftiofur, SPION and PHBV crystal are in Additional file 1.

To assess the composition and molecular architecture of the new PHBV/CEF/SPION nanoparticles and their precursors, FT-IR spectra were analyzed (Figure 3A-D). It is well-known that the ceftiofur molecule contains different functional groups that can be identified in an infrared spectrum; therefore, the most representative signals of the drug could be assigned.

From the IR spectrum, it is possible to assign signals corresponding to the carbonyl, thioester, amide and amine groups of ceftiofur at the bond-stretching frequencies of 1771, 1709, 1661 and 1661 cm^−1^, respectively. The presence of these signals helps to corroborate the formation of the drug-polymer nanoconjugate.

The spectra of the precursors of crystalline PHBV and SPION correspond to those expected for both (Additional file 1). However, some significant changes are observed in the spectrum of PHBV when the polymer is prepared in the form of nanoparticles. The most prominent bands in the spectrum of the polymer, one band centered at approximately 1530 cm^−1^ in the resonance region of N-H and O-H oscillators and another band at 1637 cm^−1^ that is assignable to the carbonyl ester stretching strongly associated with hydrogen bonding, are severely decreased. However, the normal absorption expected for the ester carbonyl group at 1737 cm^−1^ became clearly detectable in the NPs. Both features clearly indicate that the confinement of the polymer in such a small volume appears to produce a drastic loss of water.

The PHBV/CEF composite has a strong band centered at 1732 cm^-1^, possibly due to the superposition of the absorption of the ester with the carboxylic acid group of ceftiofur. Moreover, the band corresponding to N-H absorption in ceftiofur (Additional file 1) is broadened and shifted to a lower energy in the presence of the polymer; thus, this band shows a relative intensity as large as or even larger than that of the PHBV crystals. Meanwhile, the width of the band region corresponding to C = O stretching vibrations remains practically unaltered. Furthermore, the addition of SPIONs induces a decrease in the intensity of the spectrum and a well-known red shift of the bands of the C = O oscillators, whereas the band of the N-H absorption remains practically unaltered. In the PHBV/SPION/CEF nanocomposite (Figure 3C), the spectrum in these regions is better resolved in general, although the intensity is lower. Nevertheless, there is still much overlapping of vibrational bands. However, the particular frequency values of the absorption band components may at least be partially attained by analyzing the second derivative of the spectrum. Thus, an analysis of the spectral changes of ceftiofur that are associated with the formation of the nanocomposite is discussed below.

To investigate possible interactions between ceftiofur, the polymer and the metal oxide, this analysis of vibrations has been limited to the functional groups that are potentially more reactive: electrophilic or nucleophilic centers that can interact with the polymer and the metal oxide. From such a perspective, the behavior of electron donor centers (principally the carbonyl groups), as well as that of acceptor centers (e.g., the hydrogen atoms in the N-H and -O-H groups), is particularly interesting. In the polymer, the donor functionality of the carbonyl groups in the polyester is certainly the most important. However, the activity of the -COOH groups at the end of the polymer chains should also be considered. The SPION surface itself is rich in acceptor centers, coordinately unsaturated iron atoms and O-H groups, which are normally found in stabilized oxide surfaces.

In the PHBV/CEF (Figure 3D) and PHBV/SPION/CEF composites (Figure 3C), the amine and amide N-H protons of ceftiofur strongly interact with the polymer. Thus, the bands centered at 3522 and 3393 cm^−1^ in ceftiofur, which we have assigned to asymmetric and symmetric N-H stretching, are shifted to lower energy in the composite: 3421 and 3250 cm^−1^, respectively. The stretching vibration of the secondary amide N-H is found at a relatively low wavenumber, 3289 cm^−1^, in ceftiofur, indicating an association in the pristine compound; thus, the red shift observed in the composites is rather moderate. The addition of polymer affects the acidic centers of ceftiofur in a manner that does not change after the addition of SPIONs. As mentioned above, the polymer also contains some acidic centers; thus, it is expected that the addition of PHBV should also affect the carbonyl oscillators in ceftiofur. Such an effect is clearly observed in the stretching vibration of the C = O amide bond. Indeed, a red shift of approximately 14 cm^−1^ is detected in both composites.

**Figure 4:**
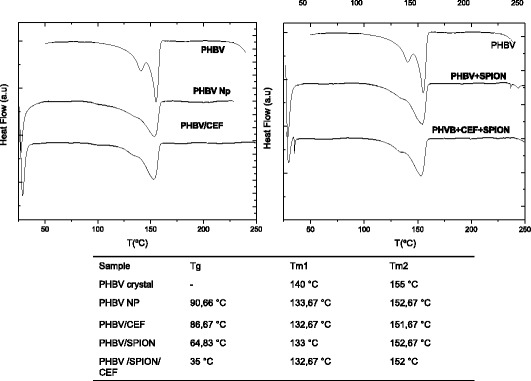
**DSC assay for PHBV nanoparticles.** The melting temperature for each sample is shown at the bottom.

**Figure 5:**
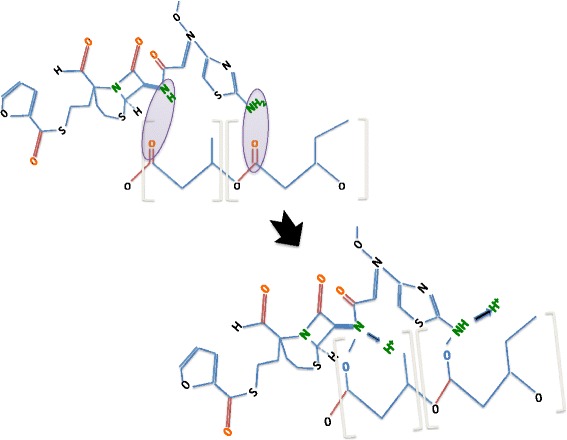
**Possible interactions between ceftiofur and the polymer in the nanoparticles according to data obtained through FTIR, UV–VIS and DSC assays.**

**Figure 6:**
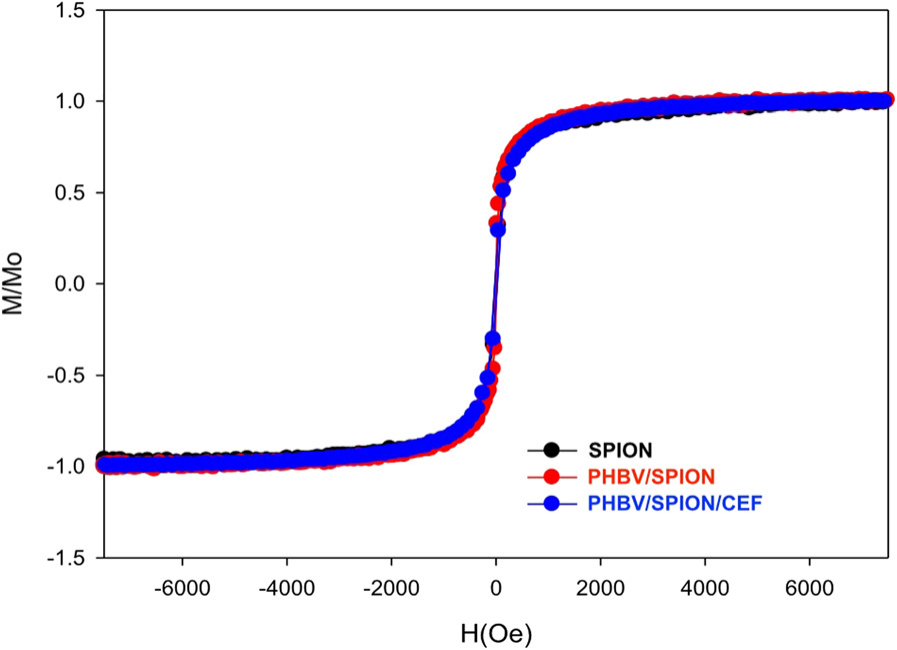
**Hysteresis loop of PHBV/SPION/CEF nanoparticles measured using a vibrating sample magnetometer (VSM).** All samples showed superparamagnetic characteristics.

To clarify the molecular structure of the PHBV nanoparticles, we analyzed these nanoparticles using DSC (Figure 4). The thermograms show a change in the crystalline behavior of the nanostructured PHBV. This fact should be attributed to the loss of degrees of freedom of the polymer due to its nanostructure; this loss could be favored by an increase in the amorphous fraction in the PHBV nanoparticles. Additionally, the PHBV-CEF nanoconjugate thermogram shows behavior that differs from that of the PHBV NP precursor because ceftiofur is incorporated inside of the cavities generated by the polymeric chains, preventing further ordering of the chains. Thus, the disorder in the polymeric chains and the amorphous fraction increases relative to the empty PHBV nanoparticles. Assays of PHBV/CEF showed that it is more amorphous than PHBV NP because ceftiofur inserted between the polymeric chains via hydrogen bridges and because ceftiofur broke some of the polymeric chains that form aldehyde groups in the valerate group of PHBV. The conformation of PHBV/SPION is similar to that of PHBV/CEF. However, PHBV/CEF/SPION has a completely different conformation (Figures 4 and 5). The crystalline fraction of PHBV/CEF/SPION is dramatically diminished; it is possible that ceftiofur and SPION interleave between polymeric chains, preventing any interaction, and this fact may explain the huge difference at 3500 cm^−1^ between Figure 3B/D and 3C. All of these results suggest a possible molecular structure for the PHBV/SPION/CEF nanoparticles. The interactions between ceftiofur and the polymer are shown in Figure 5. We have demonstrated that SPIONs and ceftiofur are incorporated into the nanoparticles and that both interact with the polymeric chains; thus, we need to further demonstrate that the SPIONs and ceftiofur do not lose their physicochemical and biological properties. Magnetic characterization using VSM measurements demonstrated the superparamagnetic behavior of the PHBV/SPION and PHBV/CEF/SPION nanoparticles (Figure 6). This fact proves that PHBV/SPION and PHBV/CEF/SPION nanoparticles have biomedical applications in MRI, hyperthermia and drug delivery to specific tissues.

**Figure 7:**
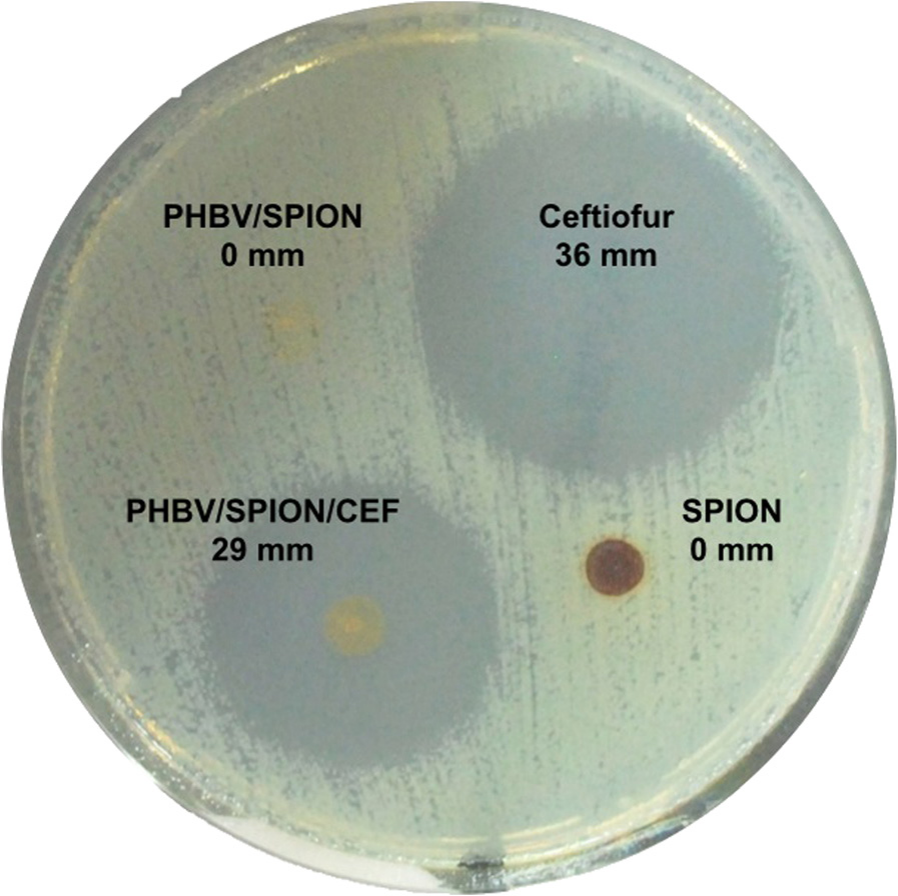
**Determination of the antimicrobial activity of PHBV nanoparticles, ceftiofur and SPIONs against** ***Escherichia coli*** **(ATCC 25922) measured using the agar diffusion method at 24 hours of incubation.**

**Figure 8:**
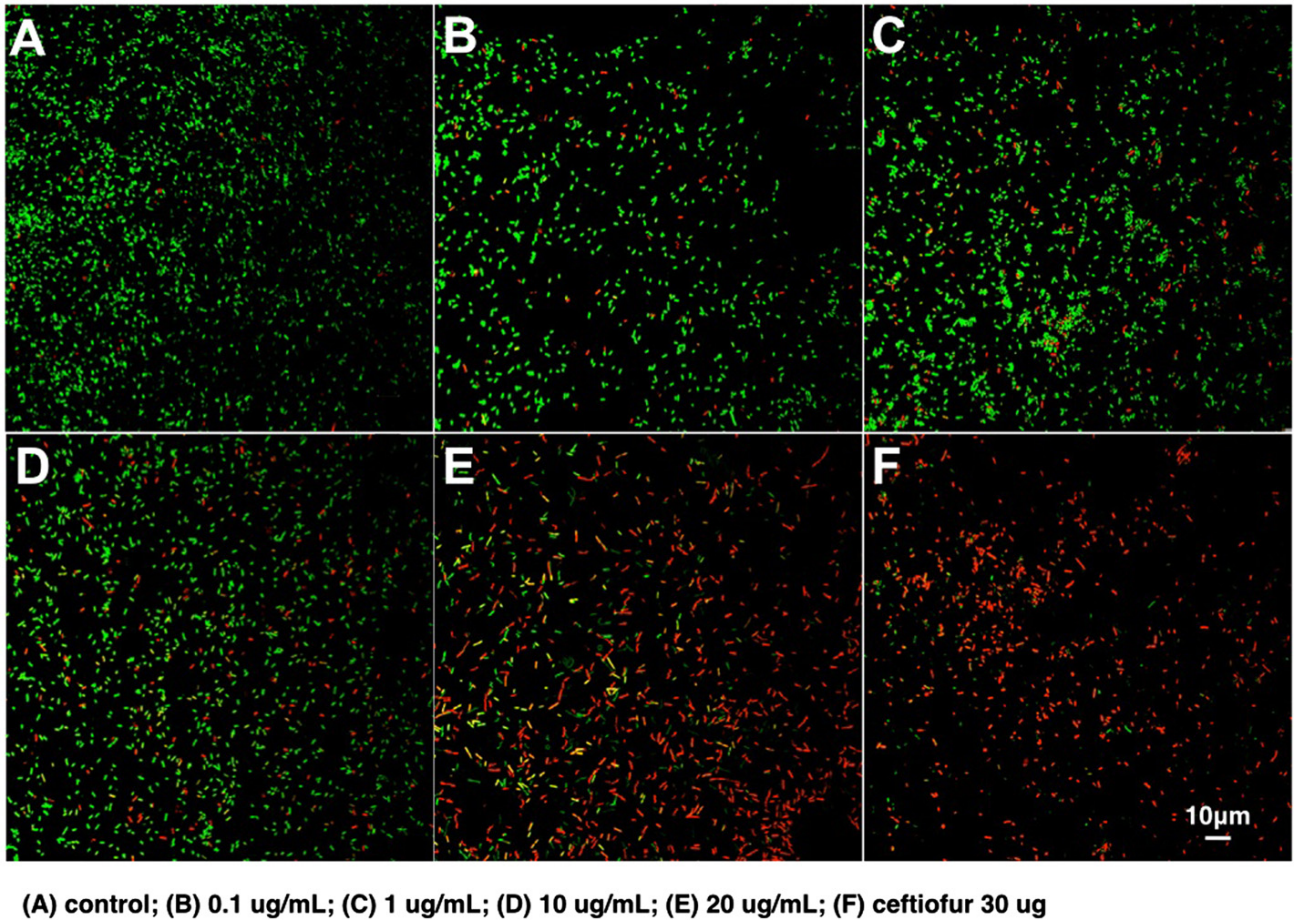
**Determination of the antibacterial activity of PHBV/SPION/CEF against** ***Escherichia coli*** **(ATCC 25922) at 24 hours of incubation using LIVE/DEAD viability assays. A)** control (without treatment), **B-E)** treatment with 0.1, 1, 10 and 20 μg/ml of PHBV/SPION/CEF nanoparticles, **F)** treatment with free ceftiofur 30 μg.

The antimicrobial activity of PHBV/CEF/SPION nanoparticles against *Escherichia coli* was measured using the agar diffusion method. These nanoparticles showed positive antibacterial activity. The inhibition halo of PHBV/CEF/SPION nanoparticles was 29 mm and that of free ceftiofur was 36 mm. PHBV nano particles without CEF did not show an inhibition halo (Figure 7). Live/dead bacterial viability assays further showed that the viability of bacteria decreased in a dose-dependent manner in samples treated with PHBV/CEF/SPION (Figure 8A-E). The positive control (30 μg/mL ceftiofur, Figure 8F) showed results similar to those of 20 μg/mL PHBV/CEF/SPION (Figure 8E).

**Figure 9:**
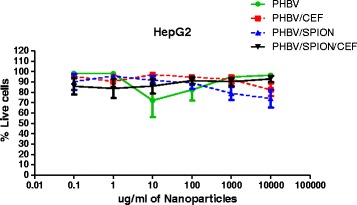
**Cytotoxicity of PHBV nanoparticles at 0.1, 1, 10, 100, 1,000 and 10,000 μg/mL against HepG2 cells using the LIVE/DEAD viability/cytotoxicity assay.**

**Figure 10:**
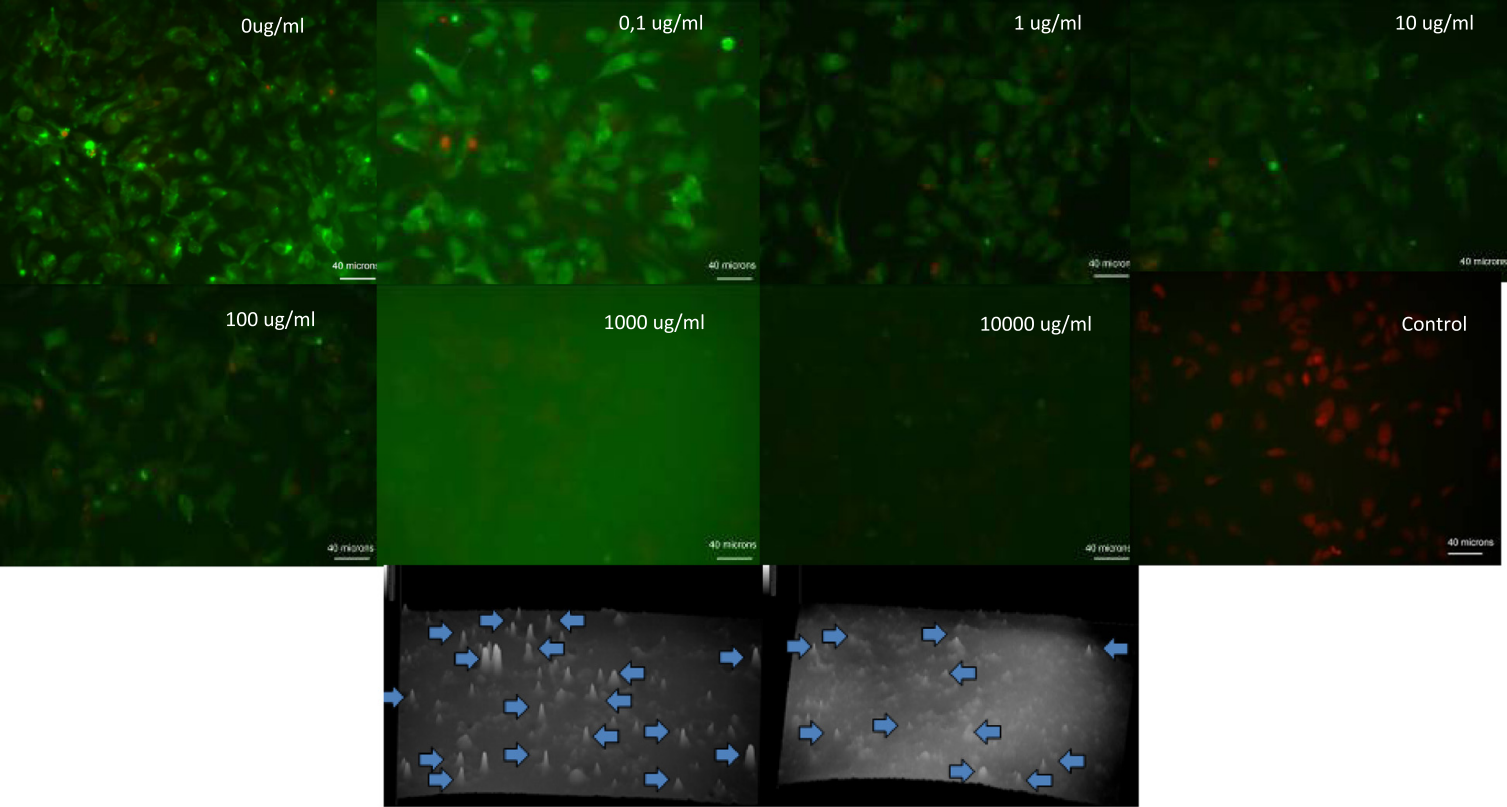
**Representative images of the cytotoxicity of PHBV/SPION/CEF against HepG2 cells using the LIVE/DEAD viability/cytotoxicity assay.** The concentration of nanoparticles is in the right corner. Bottom: fluorescence intensity plots of higher concentrations. Bar: 40 microns.

We lastly assayed the toxicological effects of 0.1 μg/mL to 10,000 μg/mL of polymeric nanoparticles in a HepG2 cell line using fluorescence microscopy (Figures 9 and 10). We used the HepG2 cell line because PHBV/CEF/SPION have potential applications in biomedicine by intravenous administration. Thus it is very important determine the toxicology in hepatic cells. Figure 9 shows a statistical analysis of the number of live and dead cells per field from a double-blind analysis of 1200 images. We show an example of these images in Figure 10 (live cells in green and dead cells in red). The background fluorescence intensity for the 1,000 μg/mL and 10,000 μg/mL samples was high because at high concentrations, the nanoparticles tended to deposit on the sample, hindering the washing. For these samples, we used fluorescence intensity plots to quantify the number of live and dead cells per field (bottom, Figure 10). Treatment with PHBV, PHBV/CEF, PHBV/SPION or PHBV/CEF/SPION nanoparticles at 0.1 μl/mL to 10,000 μg/mL did not cause significant cell death in the HepG2 cells (ANOVA p-value >0.05) (Figure 9). A more detailed analysis shows that the PHBV NP, PHBV/SPION and PHBV/CEF/SPION nanoparticles have IC_50_ > 10,000 μg/mL and that the PHBV/CEF nanoparticles have IC_50_ = 10,000 μg/ml. Statistical analysis shows that only PHBV/CEF caused significant differences in cell viability, but at doses much higher than physiological doses (10,000 μg/ml) (ANOVA and T-test < 0,05). The toxicity of PHBV/CEF is primarily caused by the high concentration of ceftiofur in the external shell of the nanoparticles.

**Figure 11:**
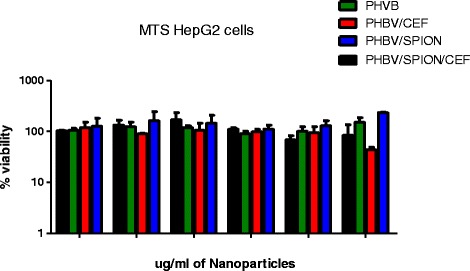
**Cytotoxicity of PHBV nanoparticles at 0.1, 1, 10, 100, 1.000 and 10.000 μg/mL against HepG2 cells using the MTS assay.**

Cell viability was measured using an MTS assay (Figure 11). The MTS assay showed that the IC_50_ of the empty PHBV, PHBV/SPION and PHBV/CEF/SPION nanoparticles was higher than 10,000 μg/mL and that the IC_50_ of PHBV/CEF nanoparticles was 10,000 μg/mL. Although the viability of the HepG2 cells decreased after treatment with PHBV/CEF nanoparticles at 10,000 μg/ml, the number of dead cells did not increase. This finding is very important because it indicates that the % viability decreased but not enough to kill the cells.

In this work, we report on the use of SPIONs in a formulation of PHBV nanoparticles loaded with ceftiofur and on the use of SPIONs as a carrier with a superparamagnetic response. The PHBV/CEF/SPION nanoparticles exhibited a spherical shape with a core-shell structure and a smooth surface. A TEM image (Figure 1) shows nanoparticles with dense black spots inside; these spots show that the SPIONs were well incorporated into the PHBV nanoparticles. The PHBV/SPION/CEF size (243.0 ± 17 nm) is optimal to have low cytotoxicity and high retention in vivo [6]. The results also show that interactions between PHBV, SPIONs and ceftiofur can change the conformation of the polymeric chains and their properties to favor the entrapment of the drug molecule. In addition, the structure of the polymer and its functionality suggest that the release process of the drug should be controlled by hydrolysis of the polymeric chains. The changes in the spectrum of ceftiofur mentioned above can be explained by considering a model in which the PHBV/CEF nanocomposite contains a SPION core. Ceftiofur is anchored to this core by a Lewis donor acceptor interaction through the amide and carboxylic C = O moieties, whereas its amine groups simultaneously interact with the polymer, forming the external shell of the nanoparticles.

Given the nature of the interactions in this nanocomposite, the system should be sensitive to changes in the pH of medium, a feature that is interesting from the perspective of the encapsulation and release of the drug.

FT-IR analysis of the products described in this work (Figure 3A-D) demonstrated the efficiency of the method used to prepare the PHBV nanoparticles and also contributed to a better understanding of the phenomena involved in this process. Indeed, the results described above show that an essential variable in this procedure is the creation of conditions that produce polymer self-aggregation. The dependence on these conditions, along with other factors, forces the polymers to a lower energy by saturating, at least partially, the ester donor moieties; this saturation occurs because the ends of the chains displace water molecules from these sites, and thus, the volume can decrease to the desired nanometer scale. Such a condition appears to fail in the presence of ceftiofur, where hydration also appears to be necessary to stabilize the excess of donor centers in the composite. The shape of the band for the PHBV/CEF nanoparticles (Figure 3D) is different and a bit more complex than that previously described for isolated ceftiofur (Additional file 1). The deconvolution of this spectrum showed a sharp band at 227 nm, which is characteristic of PHBV nanoparticles (Figure 3A), and an absorbance at approximately 337 nm that apparently corresponds to a wavelength shift in the drug-polymer ceftiofur interactions. The band assigned to the imide chromophore undergoes a relatively strong blue shift, most likely due to an inductive effect between the NH_2_ group of ceftiofur and the ester carbonyls groups of the polymer. These observations are in good agreement with the infrared spectra described below. Indeed, the observed shifts of the electronic absorption bands indicate that the polymer provides the drug with a microenvironment that differs from the pristine bulk state. In this case, these shifts are caused not only by dielectric effects but also by rather specific interactions between the donor and acceptor chromophore centers of ceftiofur and the respective groups. Along with hydration water, the electrophilic and nucleophilic groups of ceftiofur, which are available within the polymer backbone, could be involved. Due to the numerous donor centers available in the polyester (acceptor sites are restricted to only the carboxylic groups at the polymer-chain ends), it is expected that the principal contribution to the modification of the electronic configuration of ceftiofur should be the formation of hydrogen bonds between the ester carboxylic residues within the polymer backbone and the protons from the thiazolyl-amine groups. Such interactions seem to mainly be caused by the hypsochromic effects produced in both the imide and amide chromophores.

The presence of SPIONs in the nanocomposite provides a high concentration of new strong electrophilic sites. These sites are able to compete favorably to stabilize the excess of charge on the C = O groups of ceftiofur and polymer. Indeed, these groups often appear to prefer to interact with SPIONs, thus replacing polymer chain terminals as well as exogenous water molecules from the electrophilic sites in ceftiofur and the polyester. In the case of π-π* transitions in the polyenone chromophores, these types of interactions are expected to induce bathochromic effects. Of the two bands in the PHBV/CEF nanocomposite assigned above to the polymer, only the low energy broad band is detected at approximately 370 nm in the spectrum of PHBV/SPION/CEF. This red shift is also the most likely reason that the characteristic band of the polymer nanoparticles at 227 nm is not detected in the nanocomposite with SPIONs. In the case of ceftiofur confined in the nanoparticle, the amide-CO-based chromophore is supposed to be more active in the interaction with SPIONs than the chromophores based on the imide-C = N. Thus, the fact that the addition of SPIONs to the nanoparticles only moderately affects the position of the lower energy band of ceftiofur in the electronic spectrum of the PHBV/CEF nanocomposite corroborates the assignment of this absorption to the imide unit proposed above. In contrast, in the case of the amide band, the presence of SPIONs not only reverses the blue shift caused by the hydrogen bonding of the thiazolyl moiety of ceftiofur with the polymer but also further induces a red shift of approximately 15 nm with respect to the position of this band in the pristine drug. A similar (but more moderate) effect of approximately 7 nm is observed in the band assigned to the ceftiofur thiolester carbonyl, whereas the band proposed for the lactam carbonyl remains practically unaltered.

Lastly, the PHBV nanoparticles showed low cytotoxicity at high concentrations in HepG2 cells. These results indicate that PHBV is a suitable agent for entrapment antibiotics and further demonstrate its biocompatible properties.

## Conclusions

Our results indicate that these nanoparticles have potential for use in hyperthermia and could be used for drug delivery. PHBV/CEF/SPION have potential applications as a multifunctional platform because they could be used for MRI, hyperthermia applications and the release of antibiotics at an infection site with only one injection. The encapsulation of ceftiofur in PHBV nanoparticles is only one example of cephalosporin encapsulation. In theory, any cephalosporin with the same physicochemical properties as ceftiofur will behave similarly.

The size and shape of the PHBV/CEF/SPION nanoparticles can be seen in the TEM image (Figure 1). UV–VIS spectroscopy of these nanoparticles (Figure 2) shows the presence of all the desired components, and the efficiency of encapsulation was calculated using a calibration curve. Using FT-IR spectroscopy (Figure 3), we analyzed the interactions between ceftiofur, SPION and the polymer. These data were correlated with the DSC results (Figure 4) and were also used to do the model in Figure 5. The SPION activity of the PHBV nanoparticles was measured by VSM (Figure 6), and the antimicrobial activity of ceftiofur was measured by Kirby-Bauer assay (Figure 7) and microscopy (Figure 8). The toxicity of these nanoparticles was measured by microscopy in HepG2 cells. The percentage of live cells was plotted in Figure 9, and in Figure 10 an example of the microscopy photos is shown. Finally we measured the percentage of viability of the HepG2 cells by MTS (Figure 11).

The characteristics of PHBV/CEF/SPION nanoparticles give them many possible applications in biomedicine. Their superparamagnetic and antibacterial properties are very useful for treating infections in low irrigation sites, for example bone infections, because these nanoparticles allow the antibacterial dose to be localized to the site of infection. This ability can be used to reduce the doses of antibiotics, which at the same time would decrease associated adverse drug reactions. On the other hand, PHBV/CEF/SPION nanoparticles can be used in hyperthermia at infection sites, and this capability can be used to treat patients with bacterial infections associated with cancer. Vulvar, prostate, lung, gallbladder, colon, and stomach cancer have all been associated with different bacterial infections in these organs. Eliminating the infection in the cancer site can be critical to achieving tumor resection and decreasing cancer recurrence. For this reason, therapy with PHBV/CEF/SPION can be very useful for killing cancer cells and eliminating infection in the target tissue, all at the same time and with very low doses of antibiotics.

## Methods

### Preparation of PHBV nanoparticles

PHBV/CEF/SPION nanoparticles were synthesized using the water–oil-water (w_1_/o_1_/w_2_) double emulsion with solvent-evaporation method. This protocol was described previously [7,23], but in this case it was slightly modified. For the experiments, 100 μL of ceftiofur (kindly provided by Centrovet (Santiago, Chile) dissolved in methanol (100 mg/mL) (Merck, Germany) was mixed with 300 μL of a SPION suspension (magnetite, BioPAL Inc, MS, USA) (1.65 mg/mL) and was added to 1 mL of PHBV (6.25 mg/mL) (Sigma-Aldrich, St. Louis, MO, USA) dissolved in dichloromethane (DCM) (Merck, Germany). A first w_1_/o_1_ emulsion was prepared by sonication for 40 s at 100% amplitude (VCX130 ultrasonic processor, Sonics & Materials, CT, USA) over an ice bath. The water-in-oil emulsion was further emulsified under the same conditions in 2 mL of an aqueous solution of polyvinyl alcohol (PVA) (5 mg/mL) (Sigma-Aldrich, St. Louis, MO, USA). The w_1_/o_1_/w_2_ emulsion was immediately poured into a beaker that contained 30 mL of PVA solution (0.5 mg/mL) and was stirred in a hood under an exhaust fan for 16 h, allowing the solvent to evaporate. The solidified nanoparticles were harvested by centrifugation and washed with distilled water three times using an Amicon Ultra-4 centrifugal filter (Millipore, Billerica, MA, USA) with a molecular weight cut-off of 10 kDa. The PHBV/CEF/SPION were either stored at 4°C for immediate use or freeze-dried in liquid nitrogen and lyophilized for storage at −80°C for later use. We carefully synthesized empty PHBV nanoparticles (PHBV NP) using the same protocol without SPIONs or ceftiofur. PHBV/SPION nanoparticles were prepared using the same protocol but without ceftiofur, and PHBV/CEF nanoparticles were prepared using the same protocol but without SPIONs.

### Transmission electron microscopy (TEM)

The morphological examinations of the nanoparticles were performed as we described previously [23,24], using a transmission electron microscope (Phillips-TECNAI 12 BIOTWIN EM Microscope, FEI Company, Hillsboro, OR) at an acceleration voltage of 80 kV. The TEM sample was prepared by depositing 0.5 mL of the nanoparticle suspension (1.0 mg/mL) onto a 300-mesh carbon-coated copper grid (Electron Microscopy Sciences, PA, USA) that had been previously hydrophilized under UV light (Electron Microscopy Sciences, Hatfield, PA). The samples were blotted away after 20 min of incubation, and the grids were negatively stained for 5 min at room temperature with freshly prepared and sterile-filtered 2% (w/v) uranyl acetate aqueous solution (Electron Microscopy Sciences, PA, USA). Then, the grids were washed twice with distilled water and air-dried prior to imaging.

### Dynamic light scattering (DLS)

The diameter (nm) and ζ potential (mV) of the nanoparticles were analyzed by dynamic light scattering (DLS) in the Zetasizer Nano ZSP 3000 (Malvern Instruments, UK) as we described previously [12,23,24]. Each preparation was dissolved in 1 mL phosphate buffered saline (Merck, Germany) at pH 7.4, and the measurements were carried out on 3 independent formulations (batches).

### UV–VIS spectroscopy

UV–VIS spectroscopy was used to measure the lyophilized nanoparticles. The nanoparticles were analyzed using a Shimadzu spectrophotometer (UV-2450, Columbia, USA). We analyzed PHBV crystals (PHBV), empty PHBV nanoparticles (PHBV NP), PHBV/SPION nanoparticles, PHBV/CEF nanoparticles and PHBV/CEF/SPION nanoparticles. The data were analyzed using Origin software (6.0, Northampton, USA).

### FTIR spectroscopy

We lyophilized PHBV, PHBV NP, PHBV/SPION and PHBV/CEF/SPION nanoparticles. Each lyophilized nanoparticle was mixed with KBr (Sigma-Aldrich, St. Louis, MO, USA). These samples were used to make pills that were 1 cm in diameter. Each pill was analyzed using Bruker equipment (Vector 22, Hardtstrabe, Karlsruhe, Germany), and our data were collected using OPUS software (Optical user software, Hardtstrabe, Karlsruhe, Germany). Thereafter, the data were analyzed using Origin software (6.0, Massachusetts, USA).

### Differential scanning calorimetry

We lyophilized PHBV, PHBV NP, PHBV/SPION and PHBV/CEF/SPION nanoparticles. Each nanoparticle sample was weighed and then analyzed using a Mettler Toledo instrument (DSC822-E module, Barcelona, Spain) from 25°C to 250°C. The data were analyzed using Origin software (6.0, Massachusetts, USA).

### Ceftiofur entrapment efficiency

The ceftiofur entrapment efficiency was analyzed using UV–VIS spectroscopy. We obtained a calibration curve for ceftiofur and measured different concentrations of ceftiofur at 302 nm using a Shimadzu spectrophotometer (UV-2450, Columbia, USA). We then measured the lyophilized PHBV/CEF/SPION nanoparticles at 302 nm using the same equipment and calculated the amount of ceftiofur in the nanoparticles.

Alternatively, the ceftiofur entrapment efficiency was analyzed by an extraction method described previously [25]. Experimentally, 10 mg of PHBV/CEF/SPION was dissolved in 1 mL of DCM followed by the addition of 5 mL PBS buffer (pH 7.4), and it was then agitated in an orbital shaker maintained at 37°C for 24 h at 100 rpm. The ceftiofur concentration was determined by ultra-performance liquid chromatography (UPLC). Each sample was measured in triplicate, and the actual drug loading and drug encapsulation efficiency (EE) were calculated using the following equations:

$$ \begin{array}{l} Theoretical\  drug\  loading = drug\  total/\left( drug\  total+ polymer\right)\hfill \\ {} Actual\  drug\  loading = drug\  encapsulated/\left( drug\  total+ polymer\right)\hfill \\ {} Encapsulation\  efficiency=\left( actual\  drug\  loading/ theoretical\  drug\  loading\right)\times 100\%\hfill \end{array} $$

### Cell cultures

HepG2 cells (ATCC NUMBER HB-8065) were obtained from ATCC at ampule passage N° 74. These cells were cultured in MEM (Gibco, Invitrogen, Life Technologies, Grand Island, NY, USA) supplemented with 10 v/v% FBS Hyclone (Thermo Scientific, Utah, USA), 100 U/mL penicillin Hyclone (Thermo Scientific, Utah, USA), and 100 μg/mL streptomycin Hyclone (Thermo Scientific, Utah, USA) at 37°C under a humidified atmosphere of 5% CO_2_ to passage N° 76. These cells were used in all experiments.

### Antibacterial activity

Antimicrobial susceptibility was measured using the agar diffusion method as we performed previously [23] and with live/dead bacterial viability assays (Invitrogen Corporation, Carlsbad, USA). The tests were performed in triplicate against *Escherichia coli* (ATCC 25922), and we analyzed the antibacterial activity of PHBV/CEF/SPION. The agar diffusion method was conducted in accordance with the National Committee for Clinical Laboratory Standards (NCCLS) [26]. The inoculum was prepared from a Mueller-Hinton plate that had been streaked with a single colony from an initial subculture plate and incubated for 18 to 24 h. The test involved inoculating Mueller-Hinton medium and adjusting the inoculum to 1.5 × 10^8^ CFU/mL (equal to a 0.5 McFarland turbidity standard). SPIONs and PHBV/SPIONs (20 μg) were used as negative controls, and ceftiofur (30 μg) was used as a positive control. The diameter of each zone of inhibition was determined to the nearest millimeter after 24 h of incubation. In the live/dead bacterial viability assays, 1 mL of 1.5 × 10^8^ CFU/mL *Escherichia coli* and PHBV/CEF/SPION (0.1, 1, 10 and 20 μg/mL) were added to tubes with 3 mL of Mueller-Hinton liquid medium. As a negative control, we used 0.9% NaCl (Sigma-Aldrich, St. Louis, MO, USA), and as a positive control, we used ceftiofur (30 μg/mL). The live/dead bacterial viability assays were conducted according to the manufacturer’s protocol. After 24 h of incubation, each sample was mounted on a cover slip and visualized using laser scanning confocal microscopy (Axiovert 100 M Microscope, Carl Zeiss, Germany).

### Vibrating sample magnetometer (VSM)

The magnetic properties of the magnetite, PHBV/SPION and PHBV/CEF/SPION nanoparticles were performed as we described previously [7] using a vibrating sample magnetometer (VSM). The hysteresis loop was measured at 300 K as a function of an external applied field.

### Cytotoxicity assays for polymeric nanoparticles

Cell viability was examined using the MTS CellTiter 96 AQ Non-Radioactive Cell Proliferation assay (Promega, Madison, USA) as we described previously [23]. Alternatively, we used the LIVE/DEAD Viability/Cytotoxicity kit for mammalian cells (Invitrogen Corporation, Carlsbad, USA) following the manufacturer’s protocols. Cells were treated for 24 h with the following: PHBV NP, PHBV/SPION or PHBV/CEF/SPION at 0.1, 1, 10, 100, 1,000 and 10,000 μg/mL. As a negative control, the cells were treated with just vehicle (MEM medium without serum).

In the LIVE/DEAD Viability/Cytotoxicity assay, the cells were cultured in CultureSlides from BD Falcon (8 wells) with MEM supplemented 10 v/v% FBS at 37°C under a humidified atmosphere of 5% CO_2_. The seeding was 60000 cells per well, and the cells were used after 24 hours and 90% confluence. Each sample was tested in triplicate with three independent experiments, and 10 images were examined at 40× (Olympus U-RLF-T microscopy) to count the live and dead cells for each treatment. As a cytotoxicity control (100% cell death), the cells were treated for 30 minutes with 70% methanol (Merck, Germany). The statistical analyses (two-way ANOVA and T-test) were conducted using GraphPad Prism 5.0 software (California, USA).

In the MTS cell proliferation assay, the seeding was 75000 cells per well in a 96-well plate. Each sample was measured in triplicate using three independent experiments. Before the addition of MTS, the cells were washed with Hank’s medium. The cells were incubated with MTS for 1 h at 37°C under a humidified atmosphere of 5% CO_2._ The supernatant was collected in an Eppendorf tube and was centrifuged at 13000 rpm for 15 min. The measurement was performed using 90 μL of supernatant per well in a 96-well plate in an ELISA reader at 450 nm (Thermo Scientific, USA). SPIONs can interfere with the spectrometry readings. To correct these data we used the same concentration of nanoparticles in a 96-well-plate without cells. As controls we used untreated cells for 100% viability and only medium for 0% viability. The statistical analyses (two-way ANOVA and t-test) were performed using GraphPad Prism 5.0 software (California USA).

### Statistical analysis

We evaluated the significance of differences in viable and dead cells between the treatments with PHBV NP, PHBV/SPION, PHBV/CEF/SPION and without nanoparticles. We used two-way ANOVA to evaluate the differences between the treatments and the t-test to assess the differences between the treatments and the negative control. We calculated all of the statistics using GraphPad Prism 5.0 software (California, USA).

## Additional file

Additional file 1:
**FT-IR of the components of PHBV nanoparticles.** FT-IR spectra of ceftiofur, SPION and PHBV crystal. These data were used as controls to analyze the spectra in Figure 3.
