# Supplementary material for: Multifunctional polymeric nanoparticles doubly loaded with SPION and ceftiofur retain their physical and biological properties
Source: J Nanobiotechnology. 2015 Feb 13;13:14. doi: 10.1186/s12951-015-0077-5 (PMC4334767; doi:10.1186/s12951-015-0077-5)
Supplement: Additional file 1: — FT-IR of the components of PHBV nanoparticles. FT-IR spectra of ceftiofur, SPION and PHBV crystal. These data were used as controls to analyze the spectra in Figure 3. [file 12951_2015_77_MOESM1_ESM.pdf]

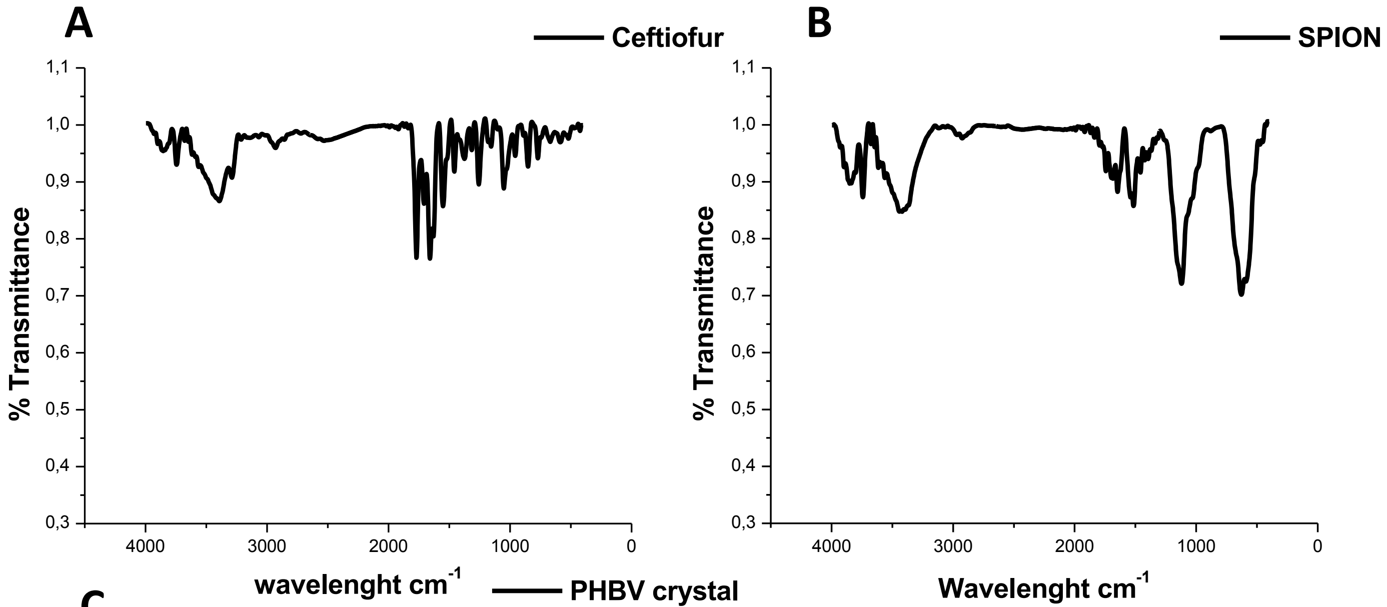

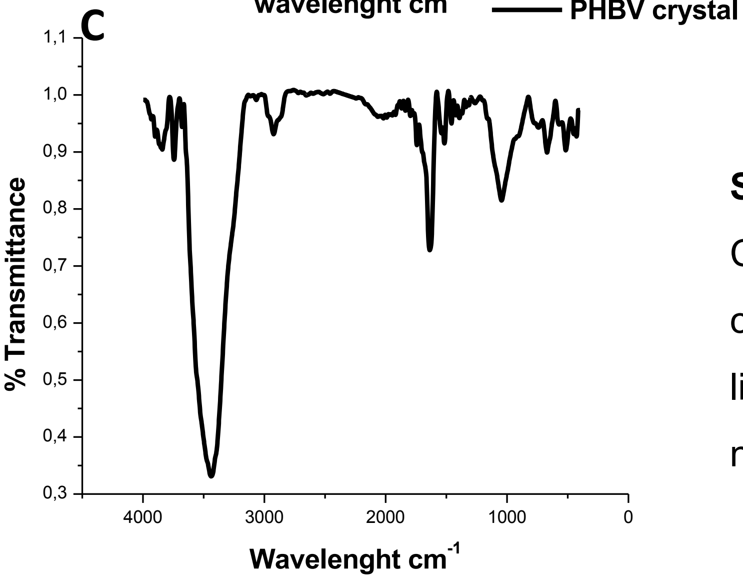

Supplemental file: FTIR for a) Ceftiofur, B)SPION and C) PHBV crystal. These samples was used like controls in FTIR for PHBV nanoparticles (Figure 4)
